# Predicting self-intercepted medication ordering errors using machine learning

**DOI:** 10.1371/journal.pone.0254358

**Published:** 2021-07-14

**Authors:** Christopher Ryan King, Joanna Abraham, Bradley A. Fritz, Zhicheng Cui, William Galanter, Yixin Chen, Thomas Kannampallil

**Affiliations:** 1 Department of Anesthesiology, Washington University School of Medicine, Saint Louis, Missouri, United States of America; 2 Institute for Informatics, Washington University School of Medicine, Saint Louis, Missouri, United States of America; 3 Department of Computer Science, McKelvey School of Engineering, Washington University in St Louis, Saint Louis, Missouri, United States of America; 4 Department of Medicine, College of Medicine, University of Illinois at Chicago, Chicago, Illinois, United States of America; 5 Department of Pharmacy Systems, Outcomes and Policy, College of Pharmacy, University of Illinois at Chicago, Chicago, Illinois, United States of America; National University of Sciences and Technology (NUST), PAKISTAN

## Abstract

Current approaches to understanding medication ordering errors rely on relatively small manually captured error samples. These approaches are resource-intensive, do not scale for computerized provider order entry (CPOE) systems, and are likely to miss important risk factors associated with medication ordering errors. Previously, we described a dataset of CPOE-based medication voiding accompanied by univariable and multivariable regression analyses. However, these traditional techniques require expert guidance and may perform poorly compared to newer approaches. In this paper, we update that analysis using machine learning (ML) models to predict erroneous medication orders and identify its contributing factors. We retrieved patient demographics (race/ethnicity, sex, age), clinician characteristics, type of medication order (inpatient, prescription, home medication by history), and order content. We compared logistic regression, random forest, boosted decision trees, and artificial neural network models. Model performance was evaluated using area under the receiver operating characteristic curve (AUROC) and the area under the precision-recall curve (AUPRC). The dataset included 5,804,192 medication orders, of which 28,695 (0.5%) were voided. ML correctly classified voids at reasonable accuracy; with a positive predictive value of 10%, ~20% of errors were included. Gradient boosted decision trees achieved the highest AUROC (0.7968) and AUPRC (0.0647) among all models. Logistic regression had the poorest performance. Models identified predictive factors with high face validity (e.g., student orders), and a decision tree revealed interacting contexts with high rates of errors not identified by previous regression models. Prediction models using order-entry information offers promise for error surveillance, patient safety improvements, and targeted clinical review. The improved performance of models with complex interactions points to the importance of contextual medication ordering information for understanding contributors to medication errors.

## Introduction

Computerized provider order entry (CPOE) systems streamline medication ordering process by creating standardized templates for the entry of legible, accurate, and complete medication orders, thereby mitigating the potential for medication errors [1–6]. By integration with electronic health record (EHR) systems, CPOE systems promote the coordination of medication ordering [7], allow real-time collaboration among care team members for medication administration, delivery and monitoring [8], and reduce the potential for misinterpretation of orders [9]. Tight coupling of the EHR with an ordering system has also led to the development of clinical decision support tools for alerting clinicians regarding improperly composed orders, clinically inappropriate orders, duplicate orders [10], wrong patient orders, and formulary non-compliance [11].

With the widespread use of CPOE systems, the volume of medications orders, and correspondingly, medication errors have increased exponentially. Rough estimates suggest that 25–30 orders are placed per inpatient admission, with nearly 4–6 additional orders per patient per day [12, 13]. Clinician interactions with CPOE systems are a source of medication errors [14–17]; errors during CPOE use account for 6–25% of detected medication errors in hospitalized patients [15]. In spite of its prevalence, much of the prior research on the causes of medication ordering errors has relied on small samples from retrospective chart reviews, clinician self-reports, analysis of malpractice claims data, and survey-based studies [18, 19]. One of the larger analysis of CPOE-based errors used manual reviews to categorize over 10,000 reported errors drawn from a national database [20], classifying the causes of such errors. Although such analyses are useful in understanding the sources of CPOE-based medication errors, these databases include limited details regarding the context of a reported error with considerable variability regarding the content of reported errors. Additionally, because of the lack of matched control (non-error) orders, they cannot be used for developing prediction models.

Previous estimates of medication errors have relied on self-reports, which often capture only the most severe errors or errors that lead to patient harm. Studies using pharmacy-based reviews are also likely to undercount errors, as orders that seem “plausible” go undetected even if they are on an incorrect patient. One approach that has received traction is the use of CPOE-integrated tools that help clinicians record self-intercepted errors within their workflow (e.g., [14, 21, 22]). Such intercepted ordering errors, although still a fraction of overall medication errors, provide considerable advantages: first, the volume of self-intercepted errors within a CPOE system is exponentially larger than manually reported errors to external incident reporting systems. Second, given that self-intercepted errors are recorded within the CPOE workflow, additional information regarding the context of the order is also easily available.

We previously described a dataset of self-intercepted errors and identified associated factors using bivariate and regression analyses [22]. That analysis identified several highly plausible risk factors for error (or interception) such as order type (e.g., inpatient, prescription) and prescriber role (e.g., physician, pharmacist, student, nurse). However, exploratory data analysis and manual stepwise modeling with massive scale data, rare outcomes, and large numbers of variables is inherently limited in its scope.

In this paper, we propose the use of machine learning (ML) approaches for characterizing the risk factors associated with medication ordering errors. Towards this end, we evaluated the performance of multiple ML methods on a large dataset of self-intercepted medication ordering errors. Such automated predictions afford opportunities for characterizing the potential causes and sources medication errors as well as allowing for automated error surveillance. We also discuss methodological advantages of using ML for predicting medication ordering errors and opportunities for using these approaches to guide patient safety efforts that are targeted towards medication orders within high-risk contexts.

## Method

A TRIPOD checklist was used for the development of the prediction model and is included in the S1 Checklist.

### Setting

Medication orders generated over a 6-year period (2006–2011) at the University of Illinois Hospital and Health Sciences System (UI Health) were studied. UI Health is a 495-bed tertiary, urban academic medical center. Orders were placed using Cerner Powerchart and Firstnet. Pharmacists or nurses entered orders based on verbal, written, or protocol orders from physicians; students entered orders requiring physician approval. Additional details of the data collection are found in a previous report [22]. This study was approved by the Institutional Review Board of Washington University in St Louis and University of Illinois at Chicago with a waiver of consent.

Medication order voiding is a CPOE-integrated function for physicians, pharmacists, nurses, and students to *self-intercept* and remove erroneous medication orders. As opposed to Medication Error Reporting Systems (MERS) reports, voiding can be performed within the ordering workflow, allowing clinicians to document errors without accessing external systems or requiring providing detailed descriptions of the error. Previous studies have shown that voided orders are a good proxy for medication errors, with voided medication orders having a 70±10% positive predictive value of being an error [14, 22]. In the paper, medication ordering errors refer to the errors identified using the voiding process. Although a field for choosing a reason for voiding existed, we have found it to be unreliable and did not use it for this analysis [14].

### Data

The outcome variable or label was order status (i.e., whether an order was voided or not). Other variables (predictors or features) included: patient demographics (race/ethnicity, sex, age), clinician type, type of medication order (inpatient, prescription, home medication by history), order date and time, and order content. Reported race was categorized into: White, Black, Hispanic, and other. Order type was classified as: inpatient (i.e., medication order for a hospitalized patient), prescriptions, and home medications by history (a non-actionable record of a medication that a patient was taking at home and was not recorded as a prescription). Time was categorized as: day (7am–5pm), night (5pm–12am), or overnight (12am–7am); Weekday was classified as: work weekday (Monday through Friday) or weekend (Saturday or Sunday). Clinician type was categorized as: physician, pharmacist, nurse, student, or other. Order content included: drug name, route, strength, volume, frequency, and dispensing unit (e.g., tablet, cap box). For each unique medication and route combination, doses were z-scored. All data were retrieved from the EHR using custom queries.

### Statistical analysis

We constructed the following models: logistic regression (LR), decision tree (DT), random forest (RF), gradient boosted decision tree (GBDT), deep embedding logistic regression (DELR), and multilayer perceptron (MLP) models. DELR is an extension of logistic regression in which each feature is fed into its own deep, narrow neural network to allow nonlinear transformation prior to entry into the logistic regression model [23]. LR, DT, RF, GBDT, and DELR were selected due to the ease of determining feature importance in each of these model architectures. Despite its generally poor interpretability, MLP was selected for its high accuracy of neural networks other applications such as image classification [24] and object detection [25].

The dataset was split into training, validation, and testing sets with a ratio of roughly 7:1:2. Because of the large sample size, repeated k-fold splits were not constructed. Missing categorical variables were added as a level of the feature. For models (such as logistic regression and MLP) that do not natively handle missing quantitative predictors, missing values were imputed with the mean observed value and a “missing value” indicator feature was concatenated. For each model, hyperparameters were searched over a grid of plausible values with tests for expansion at each endpoint, where possible. Hyperparameters that resulted in the highest area under the receiver operating characteristic curve (AUROC) in the validation set were selected and carried forward to the test set evaluation.

As the dataset was highly imbalanced (0.5% of medication orders were voided), we utilized two techniques to address class imbalance in the training set [26]. First, class weights were set as inversely proportional to their proportion. Second, we up-sampled positive training cases by 201 times, equalizing proportions. For each model architecture, both of these techniques were applied to the training set. Neither weighting nor up-sampling were performed in the validation or testing sets. The choice of class imbalance resolution technique with better performance in the validation set was used in the training model to be evaluated on the test set. That is, we treated reweighting versus up-sampling as a hyperparameter.

LR was tested with or without L2 (ridge) and L1 ratio (lasso) penalty selected from 1000, 100, 10, 1,.1 and 0,.1,.25,.5,.75, 1. LR with pairwise interactions and filtering by L1 penalty was attempted, but this classifier was difficult to optimize during training, tested very poorly, and is not reported. For DT, tree depth ranged from 3 to 8, and minimum sample split was selected from 2, 1000, 2000, 10,000, and 20,000 (a minimum sample split of 2 represents no early termination). For RF and GBDT, the number of decision trees was 100, 200, 300, 400, or 500, decision tree depth ranged from 2 to 8, and minimum sample split ranged from 2 to 20,000. For DELR, each transformation network had depth of 2 to 6 layers and width of 4 to 6 nodes. For MLP, network depth ranged from 2 to 6 layers and hidden layer width was selected from 32, 64, or 128 neurons. For both DELR and MLP, two optimizers (stochastic gradient descent with learning rate 0.1 or 0.01 and Adam optimizer with learning rate 0.001 [24]) were tested. Presence or absence of batch normalization was also tested.

Once the hyperparameters were fixed, model performance in the testing set was quantified using the AUROC metric. In highly imbalanced datasets, AUROC can sometimes be deceptive on evaluating the model performance [25]. As such, we also constructed the precision-recall curve for each model and calculated the area under the precision-recall curve (AUPRC), which is a measure of average precision. Confidence intervals for AUROC and AUPRC were created using the non-parametric logit method [27] in MatlabAUC package [28]. Each classifier was re-calibrated using isotonic regression in the validation cohort [29].

For each model, the most important features contributing to the predictions were identified. For LR, feature importance was defined by the absolute value of the regression coefficient. For DELR, feature importance was defined as the product of feature embedding value and regression coefficient. In models with deep interactions, “explainability” was approached in multiple ways. Global feature importance was assessed, for example, by permutation; however, in complex models the direction and magnitude of effect for a feature in a specific example depends on the context. For DT, feature importance was defined as the weighted entropy decrease on that feature during training phase. For RF and GBDT, feature importance was determined for the component DTs and averaged across all DTs in the model. For MLP, feature importance was quantified using backpropagation-based salience detection (integrated gradients) [30]. In this technique, the gradient of the output prediction with respect to the input feature values for each case was calculated, and the gradient with respect to each feature was averaged across the population. Integrated gradients and Shapely values were calculated for each observation, permitting local measures of feature relevance [31]. To highlight important deep interactions, we used a large global decision tree that can approximate GBDT and RF fitted surfaces [32] when appropriately supervised. We also permuted features to assess global importance to model fit. All analyses were conducted using Python version 3.7, unless otherwise specified.

## Results

The dataset included 5,804,192 orders, of which 28,695 (0.5%) were voided orders. Characteristics of the orders are shown in Table 1. Nearly two-thirds of orders were inpatient medication orders, and most orders were placed during day shifts. Overall, there were more order voiding on orders created by students (4%) or by nurses (i.e., as verbal orders, 1%).

**Table 1:** Characteristics of orders in the dataset.

Variable	Non-Voided Orders	Voided Orders
	(N = 5,775,497)	(N = 28,695)
**Age (mean, SD)**	45.5 (22.3)	46.5 (22.2)
**Age Group**		
0–19	793,672 (14%)	3,861 (13%)
20–39	1,333,142 (23%)	6,131 (20%)
40–49	826,481 (14%)	3,741 (12%)
50–59	1,127,423 (20%)	7,941 (26%)
60–69	899,144 (16%)	4,888 (16%)
70–79	540,829 (9%)	2,817 (9%)
>80	254,806 (4%)	1,296 (4%)
**Sex**		
Female	3,444,161 (60%)	16,910 (59%)
Male	2,330,629 (40%)	11,778 (41%)
**Race/Ethnicity**		
Black	2,938,539 (51%)	14,075 (49%)
Caucasian	1,217,607 (21%)	6,794 (24%)
Hispanic	729,051 (13%)	3,550 (12%)
Other	890,300 (15%)	4,276 (15%)
**Order Type**		
Normal Order	3,620,733 (63%)	17,455 (61%)
Prescription/Discharge Order	1,689,052 (29%)	5,056 (18%)
Recorded/Home Meds	465,708 (8%)	6,184 (21%)
**Shift**		
Day	3,957,683 (69%)	19,991 (70%)
Night	1,177,477 (20%)	6,148 (21%)
Overnight	640,336 (11%)	2,556 (9%)
**Title of Ordering Provider**		
Nurse	546,650 (9%)	5,684 (20%)
Pharmacist	627,963 (11%)	2,382 (8%)
Physician	3,954,439 (68%)	15,769 (55%)
Student	38,050 (1%)	1,655 (6%)
Other	608,395 (11%)	3,205 (11%)
**Normalized Drug Dose**		
Normalized Strength (mean, SD)	2.6e-4 (0.99)	-5.4e-2 (1.16)
Normalized Volume (mean, SD)	5.7e-6 (0.98)	-1.9e-3 (2.66)

SD = standard deviation

### Comparing model performance

Hyperparameters that resulted in superior AUROC in the validation set were identified. For LR, presence of L2 penalty was selected. For DT, tree depth of 8 and minimum sample split of 20,000 were selected. For RF, decision tree number of 500, decision tree depth of 8, and minimum sample split of 20000 were selected. For GBDT, decision tree number of 100, decision tree depth of 8, and minimum sample split of 2000 were selected. For DELR, transformation network depth of 4 layers, hidden layer width of 5 neurons were selected and batch normalization was enabled. For MLP, network depth of 3 layers and hidden layer width of 32 neurons were selected. For DELR and MLP, stochastic gradient descent with learning rate of 0.1 and 0.01 was selected respectively. The inverse weighting method for addressing class imbalance performed better than the up-sampling method for the DT and GBDT, while the up-sampling method performed better for the LR, RF, DELR and MLP.

**Fig 1:**
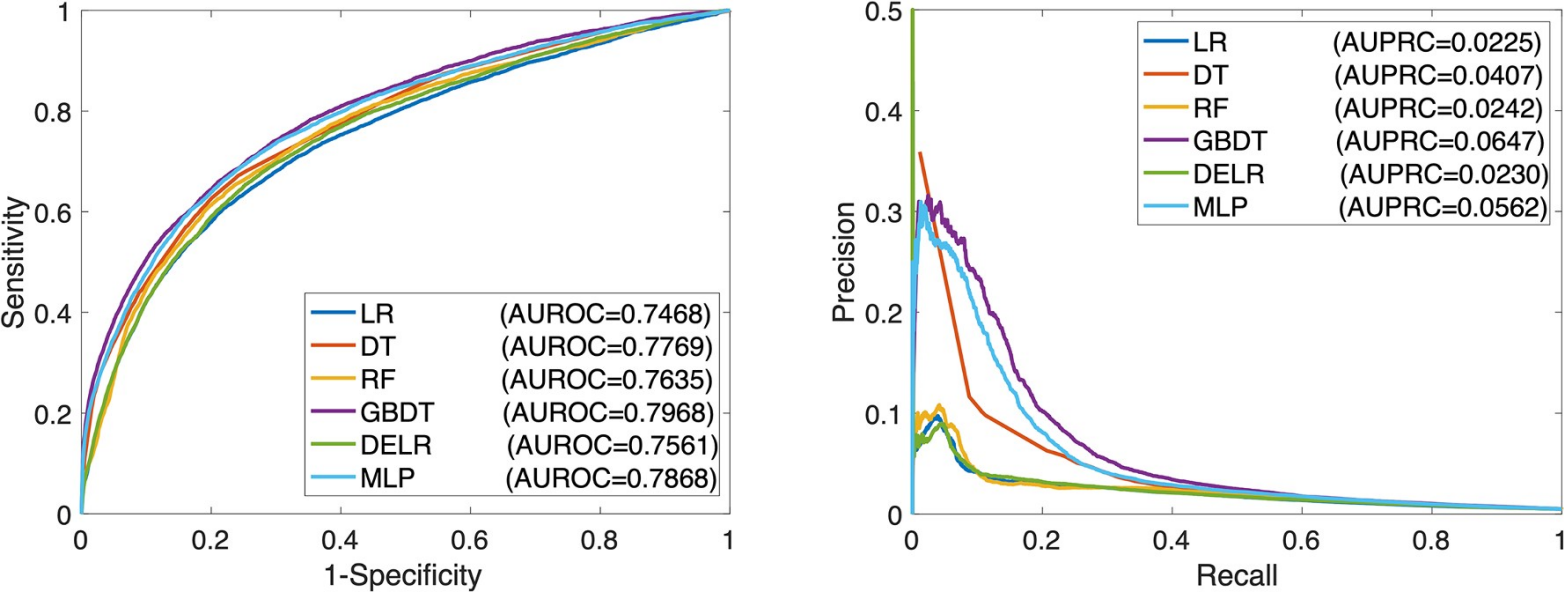
Model performance. Receiver operating characteristic curves (left panel) and precision-recall curves (right panel) demonstrating performance of each model in the testing set.

After fixing the hyperparameters, model performance in the testing set was computed (see Table 2). GBDT achieved the highest AUROC and AUPRC across all models. RF had approximately the same accuracy as DT, with marginally better AUPRC and marginally worse AUROC. LR and DELR demonstrated the poorest performance of all the models, especially in the AUPRC metric. Receiver operating characteristic and precision-recall curves for all models are shown in Fig 1. All models had acceptable calibration in the test set; the calibration curve for each model is shown in the S1 Checklist.

**Table 2:** Model performance using the test data set (*N* = 1160839).

Model	AUROC	AUPRC	NORMAL AUROC	NORMAL AUPRC
	[95% CI]	[95% CI]	[95% CI]	[95% CI]
LR	0.7468	0.0225	0.7518	0.0223
	[0.7398, 0.7536]	[0.0165, 0.0306]	[0.7430, 0.7604]	[0.0160, 0.0338]
DT	0.7769	0.0407	0.7810	0.0416
	[0.7590, 0.7938]	[0.0265, 0.0662]	[0.7581, 0.8023]	[0.0243, 0.0702]
RF	0.7635	0.0242	0.7679	0.0248
	[0.7568, 0.7701]	[0.0183, 0.0318]	[0.7594, 0.7762]	[0.0177, 0.0347]
DELR	0.7561	0.0230	0.7617	0.0239
	[0.7493, 0.7627]	[0.0172, 0.0308]	[0.7532, 0.7700]	[0.0168, 0.0339]
MLP	0.7868	0.0562	0.7888	0.0567
	[0.7803, 0.7932]	[0.0501, 0.0630]	[0.7804, 0.7969]	[0.0490, 0.0655]
GBDT	0.7968	0.0647	0.8005	0.0661
	[0.7905, 0.8030]	[0.0587, 0.0713]	[0.7925, 0.8083]	[0.0586, 0.0745]

### Model interpretation

**Fig 2:**
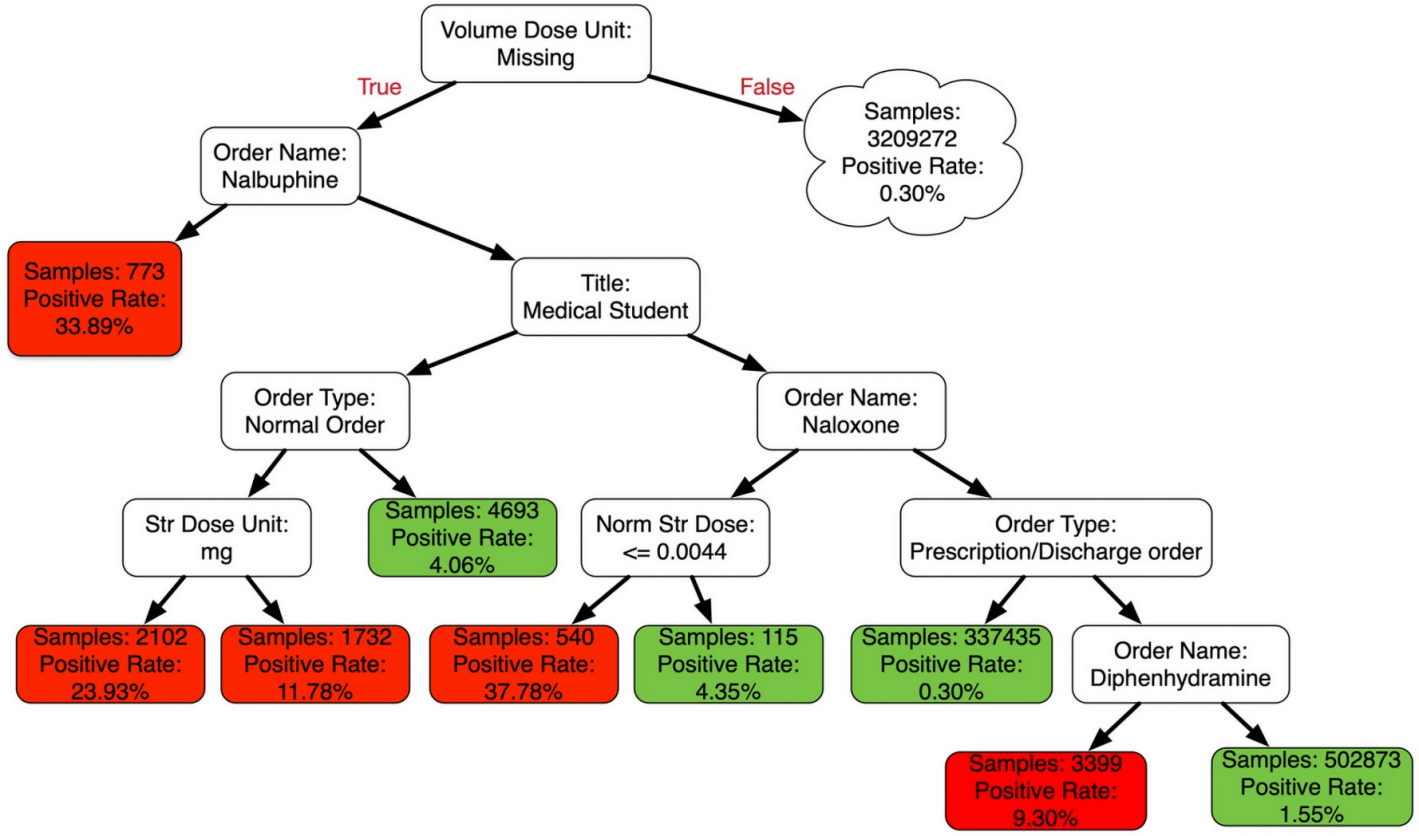
Example partial tree from the DT model showing the causal patterns for medication errors. In this tree, if the decision is false (i.e., not a voided order), take the right branch; similarly, if the decision is true (i.e., voided order), take the left branch. Red and green boxes highlight the positive rates of greater than and less than 5% respectively. Note that this is a small portion of the total tree.

We also investigated the potential for deriving meaningful explanations from the developed models. Fig 2 represents a partial decision tree for voided orders with an overall depth of 7, 54 decision nodes, and 63 leaf nodes. Displayed leaf nodes had >100 examples in the testing data; error rates within this tree varied between 0.003% to 46%. A large segment of the presented tree includes medication ordering without a value for the feature “volume dose unit” (e.g., milliliters, tablets, capsules, allowed to differ from “dispense unit” and “strength unit”). For example, the top, left-most leaf node represents orders related to the medication, nalbupine, with a volume dose unit missing or unspecified. Such orders have a high rate of being voided (~34%). This is potentially because these orders are often added outside of an order set (e.g., for pain management after surgery) or using a prespecified “order sentence.” The second node highlights orders that are entered by a medical student; for inpatient orders, 24% of such orders were likely voided.

The root node, missing volume dose unit, also highlights a contextual aspect related to the source of the medication errors. Medication orders were sometimes created using “order sentences,” where medication-related information was standardized and incorporated into an order. For instance, for the common blood pressure medication, amlodipine, an order sentence is available for a standard “10 mg, PO (“per os” or by mouth), daily.” If the clinician wanted an unusual dose or frequency, they would need to enter it as text; for example, “2.5 mg, PO, 2 AM and 1 PM.” Another situation where this occurs is when no order sentences are available, usually for rarely used medications or medications with highly varying doses. During such situations, the volume dose unit is unlikely to be defined, potentially leading to voided orders. We found that such orders with missing volume dose unit have a higher likelihood of being errors as highlighted by the sample decision tree (Fig 2).

## Discussion

To the best of our knowledge, this is the first study to utilize a large, routinely collected data set of self-intercepted medication ordering errors to forecast error status and identify associated features of these errors. Using a database of >5 million medication orders, we compared five classification algorithms for predicting medication ordering errors based primarily on the contextual features associated with a medication order. We found that GBDT was the top performing model (AUROC = 0.797) and was able to predict errors with a 10% PPV at a sensitivity of 20%. Based on a simplified classification rule using the DT algorithm, most of the voided orders had a low risk (rate ~0.3%), and about 5% with a much higher risk (rate >4%). The models highlighted the association of errors to known risk-increasing features of an order such as student-formulated orders, and to previously unknown and likely local setting-based features such as missing fields (e.g., missing volume dose unit).

The methodological approach of using ML algorithms for predicting medication errors has two potential applications. First, it is possible to identify factors associated with order entry errors that potentially represent generalizable knowledge for mitigating such errors. In Fig 2, orders that had a volume dose unit specified, most likely arising from standardized order sentences, had a lower likelihood of being voided. It is not surprising that orders that do not have a pre-defined dose unit would be associated with more errors. Such orders require more “clicks” as well as clinician recall or the need to look up dose units, fragmenting the ordering workflow. Further investigation of the use of order sentences is a promising line of investigation. Similarly, the high rate of voided student and verbal orders likely represent real and generalizable finding. Again, although not surprising, these findings have implications for training of future users. Unlike raw counts, model-based outputs are adjusted for related features, reducing the effects of confounding.

Second, ML-based medication ordering error identification offers opportunities to guide patient safety efforts that are targeted towards medication orders within high-risk contexts. “Context” has two potential meanings here. First, is the modeling interpretation of “interactions between features.” Models that did not include interactions, such as logistic regression, were less accurate, suggesting that multiple features forming a “context” is potentially required to identify medication ordering errors. In our example (Fig 2), low naloxone doses in particular were likely to be voided, suggesting that these are a confusing or easily mistaken option rather than naloxone itself as a problematic drug.

The second meaning of “context” is that many of these findings are likely situated within the context of the customized CPOE of our dataset and would neither be apparent or relevant to a multi-institutional or national database. To revisit the above example of missing volume dose unit, other CPOEs may not use the same structure to indicate “written without an order sentence/order set.” More specifically, the high rate of voided nalbuphine orders likely reflects ordering options that are not working as intended. In the limiting case, it is also possible to narrow the explanation to an individual clinician’s medication ordering characteristics [33]. Such an approach can account for the context of historical errors that are aligned with a specific clinician’s ordering characteristics or characteristics of a medication (e.g., a high-risk drug). These local findings are important to find issues in a specific CPOE implementation and user behaviors that can affect patient safety. Our methodological approach, hence, can be applied directly to hospital-scale data.

Finally, there may be value in using model-based predictions to target orders for further scrutiny. With the use of voiding function (or similar self-interception detection techniques), observed counts of errors can be a valuable source for patient safety investigations. However, in any self-report mechanism false negatives are also likely. Orders sharing characteristics with frequently voided ones (i.e., high forecasted probability) could be prospectively reviewed even if the yield is below the threshold to justify clinician-facing decision support. In our case, at a 20% sensitivity, the 10% positive predictive value implies a 20-fold decrease in the number of orders to review to discover one error. Drawing these enriched samples is a prerequisite for feasible patient safety review given that the total error rate is very low, especially for smaller clinical units within a hospital. Additionally, if units within a hospital have differential accuracy in error reporting, then forecasted error rates may be necessary to identify safety issues. Another way to understand this application is that the predicted error rate can act as a prior for a more stable estimate than the naïve reported rate in a small or noisy population. Conversely, medication ordering errors that are “easily explained” (e.g., a student order, redundant order-set orders) can be filtered out, and more complex errors investigated in greater detail for safety and quality improvement purposes.

## Conclusions and limitations

Our study has several limitations. The data was from a single academic medical center. The content findings are likely of limited generalizability. However, the methodology we have developed can be easily applied to similar datasets to identify contributors to medication ordering errors; a key advantage of ML approaches is that they are designed to be theory agnostic and account for tuning and discovery using split-sample methods. As described in our previous studies, not all voided orders were true errors. Based on chart review of voided orders in a previous study, we found that voided orders had a 60%-80% positive predictive value of being a true error [22, 34]. Approximately 22% of the self-intercepted voided orders reached the patient [34]. As voiding is optional for clinicians, certain types of orders may have been preferentially voided more frequently than others. For instance, clinicians may have preferentially voided duplicate student orders over physician orders, thus biasing the feature importance metrics. We would also like to highlight that the performance of these models is below what is needed for directly incorporating into clinical practice. For example, the false-positive rate of 90% to capture 20% of voided orders would create an unacceptable alarm burden (see Fig 1 right panel). Our dataset does not allow direct review of false-negative orders (unmarked errors); doing so would be impossible given the scale of orders that would be required. Our goal was not to replicate the domain knowledge dependent clinical decision support integrated into pharmacy systems, but to consider medication order-related factors that contribute to medication ordering errors. As such, our models did not consider details regarding the patient medical history, laboratory values, or other drug orders, which are central to most heuristic based CDS. Future studies can incorporate additional features of the medication, patient, and prescriber to improve performance. As this study was based on historical data with different configurations and order sentences, we could not verify some of the settings of the order entry system. Finally, the considered models have very different degrees of interpretability in the discussed potential applications. Logistic regression and decision trees can be directly “read off” to identify the driving factors. Although many promising efforts [35, 36] are increasing the interpretability of other classifiers, these are inherently much more complex and vulnerable to misinterpretation. Future applications will have to balance their need for interpretability and the benefits of complexity.

Medication voiding offers a promising approach for detecting, tracking and organizing medication errors. Our findings, based on applying ML models to voided orders, highlight the potential for identifying common failures of CPOE use and finding orders likely to be errors based on contextual factors. This opens new opportunities for supplementation of clinical decision support development, real-time error surveillance of efficacy of innovations in CPOE, and pragmatic patient safety efforts.

## Supporting information

S1 Checklist TRIPOD checklist & calibration curves. (DOCX)
